# Efficacy of Kinesio tape for central facial paralysis: A prospective, randomized sham-controlled, single-blinded clinical trial

**DOI:** 10.1097/MD.0000000000051043

**Published:** 2026-10-09

**Authors:** Xue Li, Jiaojiao Wang, Hua Xiao, Chenlan Shao, Shuangjiang Li, Siyi Wang, Qing Liu

**Affiliations:** a The Rehabilitation Medicine Center, People’s Hospital of Deyang City, Deyang, Sichuan, China.

**Keywords:** central facial paralysis, Kinesio tape, rehabilitation, stroke

## Abstract

**Background:**

To evaluate the safety and efficacy of Kinesio tape (KT) for managing central facial paralysis (CFP) in adults.

**Methods:**

In this randomized single-blind clinical trial, 121 patients with a 1st stroke event between June 2023 and September 2024 were randomly assigned to 1 of 3 groups (1:1:1 ratio): stimulated group (SG, KT + routine rehabilitation), sham stimulated group (SSG, KT with no tension applied + routine rehabilitation), and control group (CG, routine rehabilitation without KT). All 3 groups received routine rehabilitation therapy, with SG and SSG receiving additional KT treatment. The treatment lasted for 4 weeks, followed by a 4-week follow-up period. House-Brackmann grading scale score, Sunnybrook facial grading system, Burres–Fisch system, facial disability index, functional oral intake scale, and food residue condition were assessed at baseline (*T*0), after 4 weeks (*T*1), and 8 weeks (*T*2).

**Results:**

At *T*1, all groups showed significant improvements in House-Brackmann grading scale, Sunnybrook facial grading system, Burres–Fisch, facial disability index, and functional oral intake scale scores versus *T*0 (*P* < .05). The SG demonstrated superior therapeutic gains across all metrics except FOIS compared to SSG/CG (effect size: 1.15–2.90, *P* < .001), with benefits sustained through *T*2 (*T*1 vs *T*2 within SG: *P* > .05). Moreover, SG showed superior oral-phase efficacy (67.5% vs 45.0%/35.0% food residue condition level 0, *P* < .05). No significant differences emerged between SSG and CG at any timepoint (*P* > .05). No serious adverse events were reported.

**Conclusion:**

Our study demonstrates that tension-applied KT (25%–50% stretch) significantly improves facial symmetry (static/dynamic) and swallowing efficiency in CFP, with sustained benefits ≥4 weeks. Superior to SSG/CG, KT proves a safe adjunct therapy, enhancing motor function and quality of life in CFP rehabilitation.

## 1. Introduction

Central facial paralysis (CFP) is a common initial manifestation in stroke patients, affecting approximately 45% of cases.^[1]^ CFP typically presents as unilateral facial muscle weakness on the contralateral side of the stroke, primarily affecting the inferior facial region. This leads to drooping at the mouth corners, salivary exudation, compromised smiling ability, and limitations in both verbal communication and masticatory functions.^[2,3]^ Without effective rehabilitation, the intrinsic healing process of poststroke facial paralysis may be delayed, as evidenced by some studies.^[2]^ In patients with acute stroke, the main focus is often on limb paralysis. The wide range of clinical manifestations associated with facial dysfunction, including dysphagia, swallowing difficulties, psychosocial challenges, and aesthetic disturbances, are often overlooked.^[2]^ Current CFP treatments like neuromuscular electrical stimulation, acupuncture, and facial massage often fall short of anticipated outcomes. It is imperative to implement more effective clinical interventions to enhance treatment outcomes and minimize potential long-term consequences.

Kinesio tape (KT), a therapeutic taping technique, is an elastic adhesive tape used for treating and preventing sports injuries and various clinical conditions.^[4]^ Initially, KT was applied to patients with bone and joint injuries in clinical practice. In recent years, its application in stroke patients has been gradually augmented. Electromyographic activity has demonstrated the promoting effect of KT on muscles.^[5]^ Nevertheless, the specific effects and mechanism of action of KT remain elusive. At present, there are primarily 2 theories regarding the mechanism of KT. Firstly, KT might stimulate the exterior mechanical receptors of the skin by exerting pressure or continuous stretching upon the skin within the taped area, enhance proprioception, and further activate the regulatory mechanism within the central nervous system, manifesting an increase in muscle excitability. Secondly, as a result of the pulling effect, the space between the skin and the muscles as well as the interstitial tissue is expanded, thereby enhancing the circulation of blood and lymph fluid in the taped area.^[6,7]^ KT is cost-effective and noninvasive, providing sustained therapeutic impact throughout the day. Zhou et al disclosed that KT could enhance facial movement in stroke patients, strengthen the oral and facial muscles, and alleviate the symptom of salivation during the oral phase in stroke patients.^[8]^ However, there remains a deficiency of adequate evidence regarding the efficacy of KT for patients with CFP.

This study aimed to evaluate the efficacy and safety of KT as a therapeutic intervention for CFP, assess its short- to medium-term therapeutic effects posttreatment, and lay the groundwork for clinical rehabilitation interventions. We hypothesized that KT with tension application would yield superior clinical outcomes compared to sham or no KT interventions, primarily through enhanced proprioceptive feedback and mechanical support.

## 2. Methods

### 2.1. Study design

This prospective, randomized controlled trial with 3 arms included patients diagnosed with CFP enrolled in the study who received medical care at the People’s Hospital of Deyang City as either outpatients or inpatients between April 30, 2023 and April 30, 2024. The patients were assigned to 3 distinct groups: the stimulated group (SG), sham stimulated group (SSG), and control groups (CG), based on the intervention received. Patients provided written informed consent, and the study was approved by the Medical Ethics Committee of People’s Hospital of Deyang City (2023-04-030-K01) and registered in the Chinese Clinical Trial Registry (ChiCTR2300071915).

### 2.2. Participants

We adult patients diagnosed with stroke-related CFP at Deyang People’s Hospital were prospectively enrolled in the study. The inclusion criteria were: unilateral cerebral ischemic or hemorrhagic stroke lesions confirmed by computed tomography or magnetic resonance imaging; all participants were enrolled within 6 months of their 1st stroke event, as confirmed by medical records; stroke-related CFP; clear cognition and ability to cooperate with the evaluator for assessment; and age 18 years or older. The exclusion criteria were: severe allergy to the KT tape used in this trial; other neurological diseases; severe corneal infection (corneal ulceration or blindness); and refusal to participate in the study. The study termination criteria were: major diseases relapsed throughout the research period; a serious allergic reaction occurred; the patient requests withdrawal; and severe infection of the cornea.

### 2.3. Randomization and blinding

Patients were randomly assigned to the SG, SSG, and CG in a 1:1:1 ratio using a computer-generated random number table. For serious adverse events during the trial, emergency unblinding was conducted following relevant procedures. The assessment staff were blinded to the main study design and group allocation.

### 2.4. Sample size calculation

The primary endpoint of this study was the recovery of facial nerve function. The sample size was calculated using the House-Brackmann grading scale (HBGS). Based on previous studies,^[9]^ which reported a post-intervention HBGS score of 3.5 ± 1.2, we assumed a clinically significant difference of 1 point (Δ = 1) between groups. With α = 0.05 and 80% power (β = 0.2), the calculated sample size was 35 participants per group (n = 35 × 3 = 105 total). To account for a potential 15% attrition rate in this longitudinal rehabilitation study, the final target sample size was adjusted to 121 participants (105 × 1.15 = 120.75, rounded to 121). Participants were allocated as follows to maintain a balanced 1:1:1 ratio while accommodating practical enrollment constraints: 41 participants in SG, 40 in SSG, and 40 in CG (N = 121).

### 2.5. Interventions

All participants received routine rehabilitation therapy, including daily 20-minute acupuncture sessions targeting facial nerve points and 20-minute neuromuscular electrical stimulation applied to the orbicularis oris, frontalis, and zygomaticus muscles. Adherence to both treatments remained consistent throughout the study period. In addition to the conventional rehabilitation protocol, participants in the SG underwent facial KT intervention. The taping procedure utilized 3 I-shaped KT strips (2.5 cm width, KT tape) through a combined approach based on spatial correction, functional correction, and neurostimulation techniques, implemented as follows: based on the spatial correction technique, the 1st longer strip was applied with 25% tension over the preauricular facial nerve region, with tension-free anchoring at the temporal and mandibular branches; based on the functional correction technique, the 2nd short strip was anchored at the preauricular site and extended with 50% tension toward the affected oral commissure along the contraction vector of the zygomaticus major muscle, dynamically simulating physiological muscle contraction to facilitate facial movements (e.g., smiling); and based on the neurostimulation technique, the 3rd short strip was anchored at the preauricular area and applied with 50% tension along the zygomatic branch of the facial nerve to enhance neural pathway activation and stabilize midfacial symmetry.^[10]^ Participants in the SSG received identical tape placement but maintained 0% tension throughout to eliminate mechanical effects, preserving only cutaneous contact for placebo control. This design effectively differentiated the physical therapeutic effects of KT from psychophysiological influences. The CG participants strictly adhered to the standardized conventional rehabilitation protocol, excluding any exogenous physical interventions. CG participants, serving as the control group, received no KT intervention and followed only the standardized rehabilitation regimen to ensure baseline treatment consistency. All KT applications were standardized and administered by a single physical therapist specialized in neurorehabilitation. Tapes were replaced daily and worn continuously for 24 hours (Fig. 1). This protocol minimized inter-operator variability.

**Figure 1.**
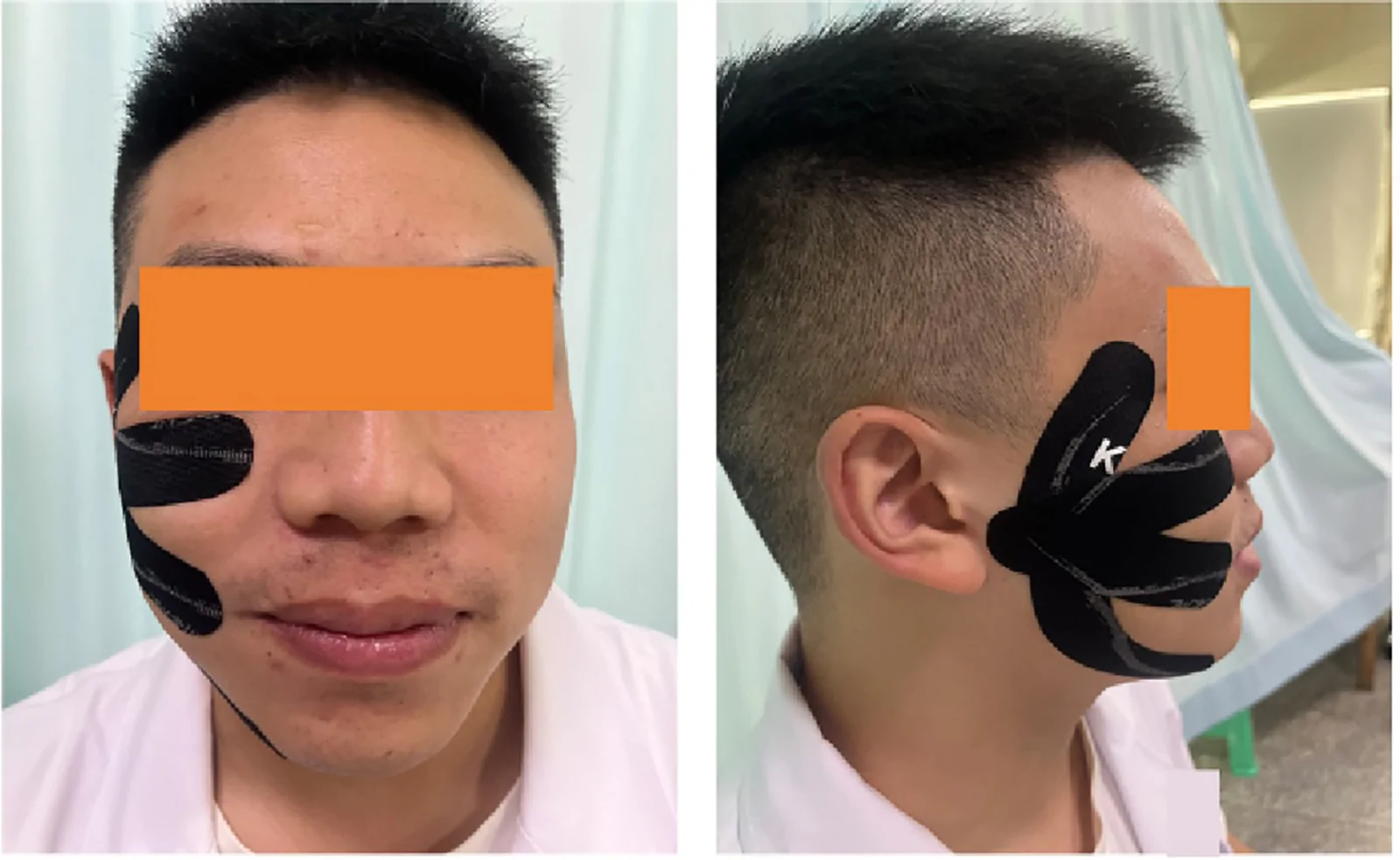
A schematic representation depicting the application of KT to the facial region of the participant. KT = Kinesio tape.

### 2.6. Outcome assessment

#### 2.6.1. Facial static and dynamic functions

The primary endpoint was assessed using HBGS,^[11–13]^ a 1- to 6-point scale, with higher grades indicating greater severity. Secondary endpoints included the Sunnybrook facial grading system (SFGS)^[11,14,15]^ and the Burres–Fisch (BF) system.^[16–18]^ The SFGS scoring system ranges from 0 to 100, with higher scores indicating superior facial nerve function. First, the physician evaluates the SFGS_rest scores, which include the symmetry of the eye, cheek (nasolabial fold), and mouth at rest. Each category is scored from 0 to 2, with a sum-weighted factor of 5. The maximum achievable score is 20, with a higher score indicating poorer static symmetry. Next, the physician assesses SFGS_move using a 5-point scale to evaluate facial movements across 5 standard expressions. Each movement is scored from 1 to 5, with 1 denoting the absence of movement and 5 indicating normal movement. The cumulative scores are then multiplied by a factor of 4, resulting in a maximum score of 100. Higher scores indicate improved dynamic symmetry. In the 3rd stage (SFGS_synkinesis), the physician evaluates synkinesis severity using a 3-point scale (0 = none, 1 = mild, 2 = moderate, and 3 = severe) for each of the 5 expressions. The total synkinesis score is then multiplied by a weighted factor of 1. The final synkinesis score (SFGS_total) is calculated as SFGS_move − SFGS_rest − SFGS_synkinesis.^[11,14,15]^ The assessment of BF^[16–18]^ involves evaluating resting symmetry (BF_rest) and measuring voluntary movement symmetry (BF_move). The final score (BF_total) is the sum of these 2 components, with higher scores indicating greater facial paralysis severity.

#### 2.6.2. Quality of life

The facial disability index (FDI)^[19,20]^ assessed the impact of CFP on patients’ quality of life, including physical disability (FDIp, 5 questions, 0–30 scale; higher scores indicate better functionality) and social function (FDIs, 5 questions, 0–40 scale; lower scores suggest a reduced impact of facial paralysis on social activities and improved social functioning ability among patients).

#### 2.6.3. Dysphagia

Dysphagia severity was evaluated using the functional oral intake scale (FOIS)^[21,22]^ (7-level scale; higher scores indicate improved swallowing function) and food residue condition (FRC), classified as 0 (none), 1 (mild), 2 (moderate), or 3 (severe). Adverse events were monitored to assess KT safety in CFP patients.

### 2.7. Statistical analysis

All statistical analyses were conducted using SPSS version 26.0 (IBM Corp.), encompassing data cleaning, descriptive statistics, and inferential analyses. Continuous variables were expressed as mean ± standard deviation when normality assumptions were satisfied (assessed via Shapiro–Wilk test, *P* > .05), or median with interquartile range for non-normally distributed data, while categorical variables were summarized as frequencies and percentages. Baseline comparisons employed Pearson chi-square test (or Fisher exact test for small cell counts) for categorical variables and 1-way analysis of variance (ANOVA) (with Levene test for homogeneity of variance) or Kruskal–Wallis *H* test for nonparametric continuous variables to assess intergroup differences among SG, SSG, and CG. Intragroup comparisons between pre-intervention (week 0) and post-intervention (week 4) utilized paired *t* tests for parametric data or Wilcoxon signed-rank tests for nonparametric data. Intergroup treatment effects were evaluated using 1-way ANOVA followed by least significant difference post hoc pairwise comparisons (or Kruskal–Wallis *H* test with Dunn–Bonferroni correction for nonparametric data). Longitudinal changes across 3 time points (week 0, week 4, and week 8) were analyzed via 2-way repeated-measures ANOVA incorporating group (SG/SSG/CG) and time (baseline/post-intervention/follow-up) as factors, with Greenhouse–Geisser adjustment applied if sphericity assumptions were violated (Mauchly test, *P* < .05). A 2-tailed *P* < .05 defined statistical significance.

## 3. Results

This study screened 229 patients with CFP, of whom 108 did not meet the inclusion criteria. A total of 121 eligible participants underwent baseline assessment and were randomly assigned to 3 groups: SG (n = 41), SSG (n = 40), and CG (n = 40). During the intervention, 1 participant in the SG discontinued due to a recurrent stroke. Thus, 120 participants (40 per group) ultimately completed the full intervention protocol. Their demographic and clinical baseline characteristics are presented in Table 1. Statistical analysis revealed that, aside from a difference in the distribution of facial palsy side (*P* = .045), no other baseline characteristics showed significant differences among the groups (*P* > .05), indicating that the groups were well-balanced and comparable. All 120 participants who completed the intervention were included in the intention-to-treat analysis for the primary efficacy outcomes (Table 2). During the follow-up period, 4 participants were lost to follow-up: 1 from the SG (transferred to a local hospital) and 3 from the SSG (2 were lost to contact and 1 transferred to a local hospital). No losses occurred in the CG. Consequently, 116 participants (SG: n = 39; SSG: n = 37; CG: n = 40) completed the entire study from intervention through follow-up. Their data formed the per-protocol analysis set used to assess sustained efficacy outcomes during follow-up (Fig. 2).

**Table 1:** Baseline characteristics of the 3 groups.

Characteristics	SG (n = 40)	SSG (n = 40)	CG (n = 40)	*P* value†
Sex (M/F)	28/12	29/11	25/15	.606
Age (yr)*	57.50 ± 12.38	60.85 ± 10.10	59.10 ± 11.44	.420
Stroke type (hemorrhage/infarction)	18/22	18/22	18/22	1.000
Facial paralysis side, (left/right)	27/13	16/24	20/20	.045*
Disease duration (d)	29.38 ± 27.19	36.9 ± 33.10	33.80 ± 30.07	.540
Hypertension (yes/no)	34/6	39/1	37/3	.126
Diabetes (yes/no)	9/31	13/27	5/35	.101
Hyperlipemia (yes/no)	6/34	3/37	7/33	.392
FOIS*	5.15 ± 2.12	5.08 ± 1.98	5.05 ± 2.25	.976
Food residue (no residue/residue)	1/39	1/39	1/39	.868
HBGS*	4.37 ± 0.70	4.35 ± 0.62	4.25 ± 0.71	.683
BF_rest*	3.10 ± 0.84	3.28 ± 0.75	3.03 ± 0.77	.349
BF_move*	10.85 ± 5.53	10.38 ± 3.91	9.18 ± 3.56	.221
BF_total*	13.90 ± 6.13	13.60 ± 4.35	12.23 ± 4.13	.276
SFGS_rest*	13.50 ± 4.96	13.38 ± 4.58	12.78 ± 4.66	.751
SFGS_move*	71.78 ± 14.36	72.20 ± 10.98	75.10 ± 11.08	.420
SFGS_total*	58.90 ± 18.74	58.65 ± 14.32	62.90 ± 14.68	.416
FDIP*	68.63 ± 17.83	66.25 ± 17.68	64.50 ± 19.57	.603
FDIS*	43.43 ± 12.53	47.75 ± 15.00	42.20 ± 11.74	.145

BF = Burres–Fisch grading system, CG = control group, FDIp = facial disability index scale-physical function, FDIs = facial disability index scale-social function, FOIS = functional oral intake scale, HBGS = House-Brackmann grading scale, SFGS = Sunnybrook facial grading system, SG = stimulated group, SSG = sham stimulated group.

* Indicates the data have a parametric distribution presented as mean ± SD.

† Indicates 1-way analysis of variance or chi-square test.

* Indicates statistical difference (*P* < .05).

**Table 2:** The changes of the 3 groups of outcome indicators before and after the intervention.

Outcomes	Intervention	Pre (baseline)	Post (4th week)	ES (Cohen *d*) 95% CI	*t*	*P*1
HBGS	SG (n = 40)	4.37 ± 0.70	2.35 ± 0.66^a^	2.90 (2.19–3.61)	18.36	<.001
	SSG (n = 40)	4.35 ± 0.62	3.55 ± 0.60^b^	1.55 (1.08–2.00)	9.80	<.001
	CG (n = 40)	4.25 ± 0.71	3.63 ± 0.70^b^	1.28 (0.85–1.69)	8.06	<.001
	*F*	0.379	47.555			
	*P*2	0.485	<0.001			
BF_rest	SG (n = 40)	3.10 ± 0.84	1.13 ± 0.76^a^	2.38 (1.76–2.98)	15.02	<.001
	SSG (n = 40)	3.28 ± 0.75	2.38 ± 0.74^b^	1.27 (0.85–1.68)	8.03	<.001
	CG (n = 40)	3.03 ± 0.77	2.43 ± 0.81^b^	1.21 (0.80–1.61)	7.65	<.001
	*F*	1.062	36.518			
	*P*2	0.349	<0.001			
BF_move	SG (n = 40)	10.85 ± 5.53	3.88 ± 3.33^a^	1.68 (1.20–2.16)	10.65	<.001
	SSG (n = 40)	10.38 ± 3.91	8.15 ± 3.20^b^	1.30 (0.87–1.71)	8.20	<.001
	CG (n = 40)	9.18 ± 3.56	7.60 ± 3.10^b^	1.15 (0.74–1.54)	7.24	<.001
	*F*	1.528	20.971			
	*P*2	0.221	<0.001			
BF_total	SG (n = 40)	13.90 ± 6.13	5.00 ± 3.76^a^	1.91 (1.38–2.43)	12.09	<.001
	SSG (n = 40)	13.60 ± 4.35	10.55 ± 3.52^b^	1.41 (0.96–1.84)	8.88	<.001
	CG (n = 40)	12.23 ± 4.13	10.03 ± 3.74^b^	1.36 (0.92–1.76)	8.59	<.001
	*F*	1.302	27.792			
	*P*2	0.276	<0.001			
SFGS_rest	SG (n = 40)	13.50 ± 4.96	4.90 ± 3.06^a^	1.81 (1.30–2.32)	11.47	<.001
	SSG (n = 40)	13.38 ± 4.58	9.25 ± 4.17^b^	1.50 (1.04–1.95)	9.50	<.001
	CG (n = 40)	12.78 ± 4.66	9.75 ± 4.78^b^	0.95 (0.57–1.32)	6.00	<.001
	*F*	0.288	17.161			
	*P*2	0.751	<0.001			
SFGS_move	SG (n = 40)	71.78 ± 14.36	92.35 ± 4.74^a^	−1.67 (−2.14 to −1.18)	−10.54	<.001
	SSG (n = 40)	72.20 ± 10.98	80.90 ± 10.91^b^	−1.18 (−1.58 to −0.77)	−7.44	<.001
	CG (n = 40)	75.10 ± 11.08	80.00 ± 10.91^b^	−1.10 (−1.49 to −0.70)	−6.95	<.001
	*F*	0.874	21.826			
	*P*2	0.420	<0.001			
SFGS_total	SG (n = 40)	58.90 ± 18.74	87.45 ± 6.24^a^	−1.83 (−2.34 to −1.32)	−11.60	<.001
	SSG (n = 40)	58.65 ± 14.32	71.15 ± 13.26^b^	−1.33 (−1.76 to −0.90)	−8.42	<.001
	CG (n = 40)	62.90 ± 14.68	71.33 ± 14.27^b^	−1.17 (−1.56 to −0.76)	−7.37	<.001
	*F*	0.885	25.140			
	*P*2	0.416	<0.001			
FDIp	SG (n = 40)	68.63 ± 17.83	95.13 ± 6.15^a^	−1.66 (−2.13 to −1.17)	−10.49	<.001
	SSG (n = 40)	66.25 ± 17.68	79.38 ± 15.28^b^	−1.63 (−2.10 to −1.15)	−10.30	<.001
	CG (n = 40)	64.50 ± 19.57	76.25 ± 16.00^b^	−1.19 (−1.60 to −0.78)	−7.55	<.001
	*F*	0.508	23.285			
	*P*2	0.603	<0.001			
FDIs	SG (n = 40)	43.43 ± 12.53	29.20 ± 9.01^a^	1.15 (0.74–1.54)	7.26	<.001
	SSG (n = 40)	47.75 ± 15.00	40.83 ± 15.94^b^	0.95 (0.57–1.32)	5.98	<.001
	CG (n = 40)	42.20 ± 11.74	37.45 ± 13.68^b^	0.61 (0.27–0.94)	3.84	<.001
	*F*	1.962	8.218			
	*P*2	0.145	<0.001			
FOIS	SG (n = 40)	5.15 ± 2.12	6.78 ± 0.70	−0.88 (−1.24 to −0.51)	−5.56	<.001
	SSG (n = 40)	5.08 ± 1.98	6.35 ± 1.10	−0.93 (−1.29 to −0.55)	−5.86	<.001
	CG (n = 40)	5.05 ± 2.25	6.40 ± 1.34	−0.84 (−1.20 to −0.47)	−5.30	<.001
	*F*	0.02	1.86			
	*P*2	0.98	0.16			

All the data have a parametric distribution presented as mean ± SD; paired *t* tests were employed for intragroup comparisons (*P*1), while 1-way analysis of variance was utilized for intergroup comparisons (*P*2). According to the letters a and b, 3 groups are used to indicate statistical differences. If 2 groups have the same letter (b, b), it means there is no statistical difference between them; while if the letters between 2 groups are different (a, b), it implies that there is a significant statistical difference between them (*P* < .05).

BF = Burres–Fisch grading system, CG = control group, ES = effect size, FDIp = facial disability index scale-physical function, FDIs = facial disability index scale-social function, FOIS = functional oral intake scale, HBGS = House-Brackmann grading scale, SFGS = Sunnybrook facial grading system, SG = stimulated group, SSG = sham stimulated group.

**Figure 2.**
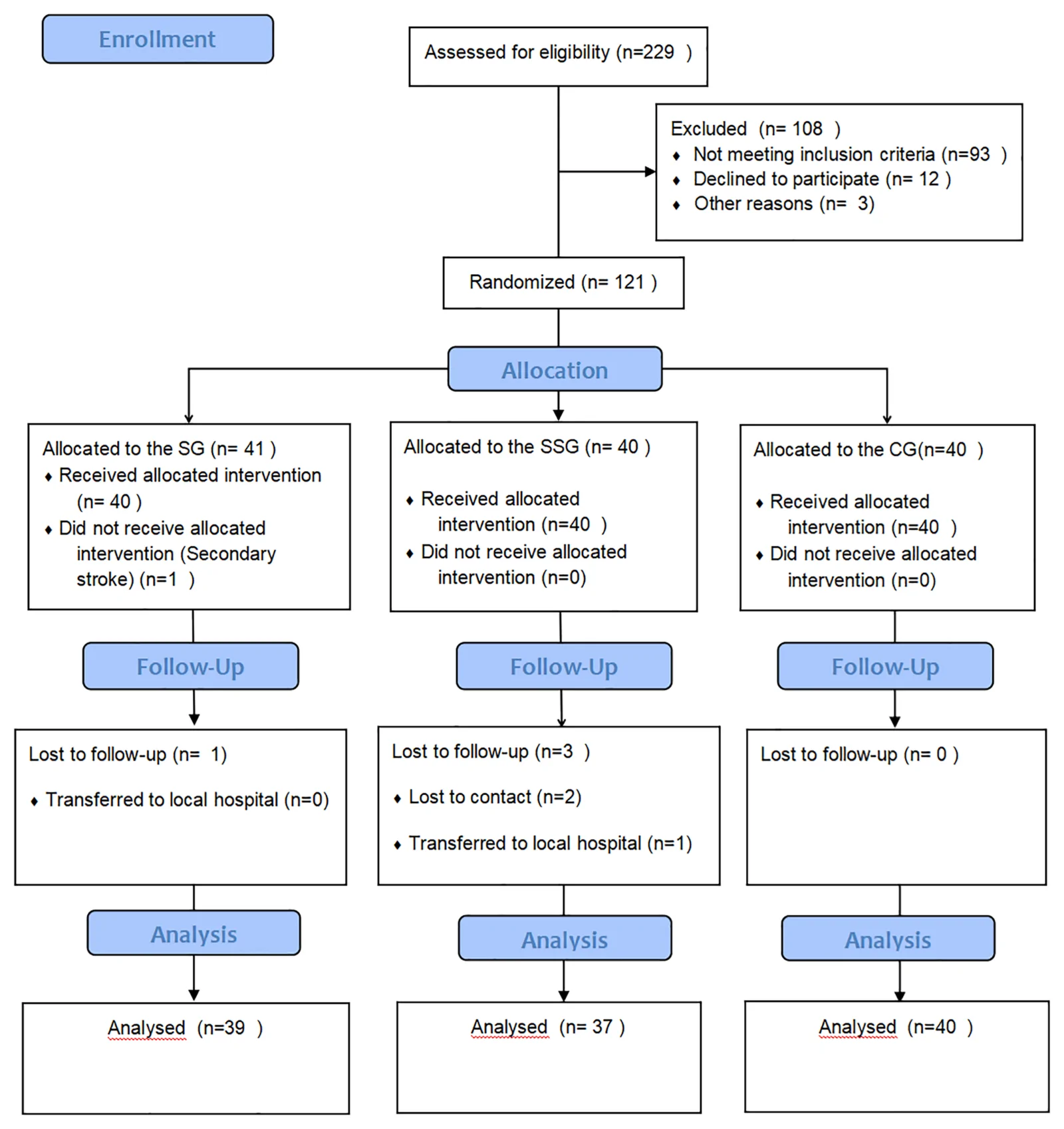
Flow of participants in a randomization trial of subjects with central facial paralysis. CG = control group, SG = stimulated group, SSG = sham stimulated group.

### 3.1. Facial static and dynamic functions

The participants demonstrated improved dynamic and static facial symmetry before and after the intervention. Figure 2 shows the facial changes observed in the 2 patient groups before and after treatment. The results indicated no significant differences in static and dynamic facial functions (HBGS, BF_rest, BF_move, BF, SFGS_rest, SFGS_move, and SFGS_total) among the 3 groups before treatment (*T*0) (Table 1). However, after the 4-week intervention (*T*1), statistically significant improvements were observed in all 3 groups compared to pretreatment (Table 2). Notably, the effect size in the SG showed the most significant improvements compared to the SSG and CG groups (Table 2). Additionally, we monitored changes in facial function 4 weeks posttreatment discontinuation to evaluate the sustainability of the intervention’s effects. To evaluate the sustainability of therapeutic effects, longitudinal analyses of facial function changes were conducted at 4 weeks post-intervention (*T*1) and 8-week follow-up (*T*2). Two-way repeated-measures ANOVA demonstrated significant main effects of time (all *F* ≥ 189.742, *P* < .001, partial η^2^ = 0.627–0.802), Group (all *F* ≥ 6.563, *P* ≤ .002, partial η^2^ = 0.104–0.294), and time × group interactions (all *F* ≥ 25.775, *P* < .001, partial η^2^ = 0.313–0.552) across all outcomes (Table S1, Supplemental Digital Content 1). Simple effects analysis revealed no baseline differences (*T*0) among groups (*P* > .05 for all metrics), whereas SG exhibited superior improvements in HBGS, BF_rest, BF_move, BF_total, SFGS_rest, SFGS_move, and SFGS_total compared to SSG and CG at both *T*1 and *T*2 (*P* < .05), with no significant differences between SSG and CG (*P* > .05) (Table S1, Supplemental Digital Content 1). After standardizing the baseline values by calculating the differences between these values and weeks 4 and 8, SG’s therapeutic benefits remained stable from *T*1 (week 4) to *T*2 (week 8) (within-group *T*1 vs *T*2: *P* > .05), consistently outperforming SSG and CG (*P* < .001 for all intergroup comparisons) (Fig. 3). These findings indicate that KT combined with routine rehabilitation not only rapidly enhances facial symmetry but also sustains efficacy for at least 4 weeks post-intervention.

**Figure 3.**
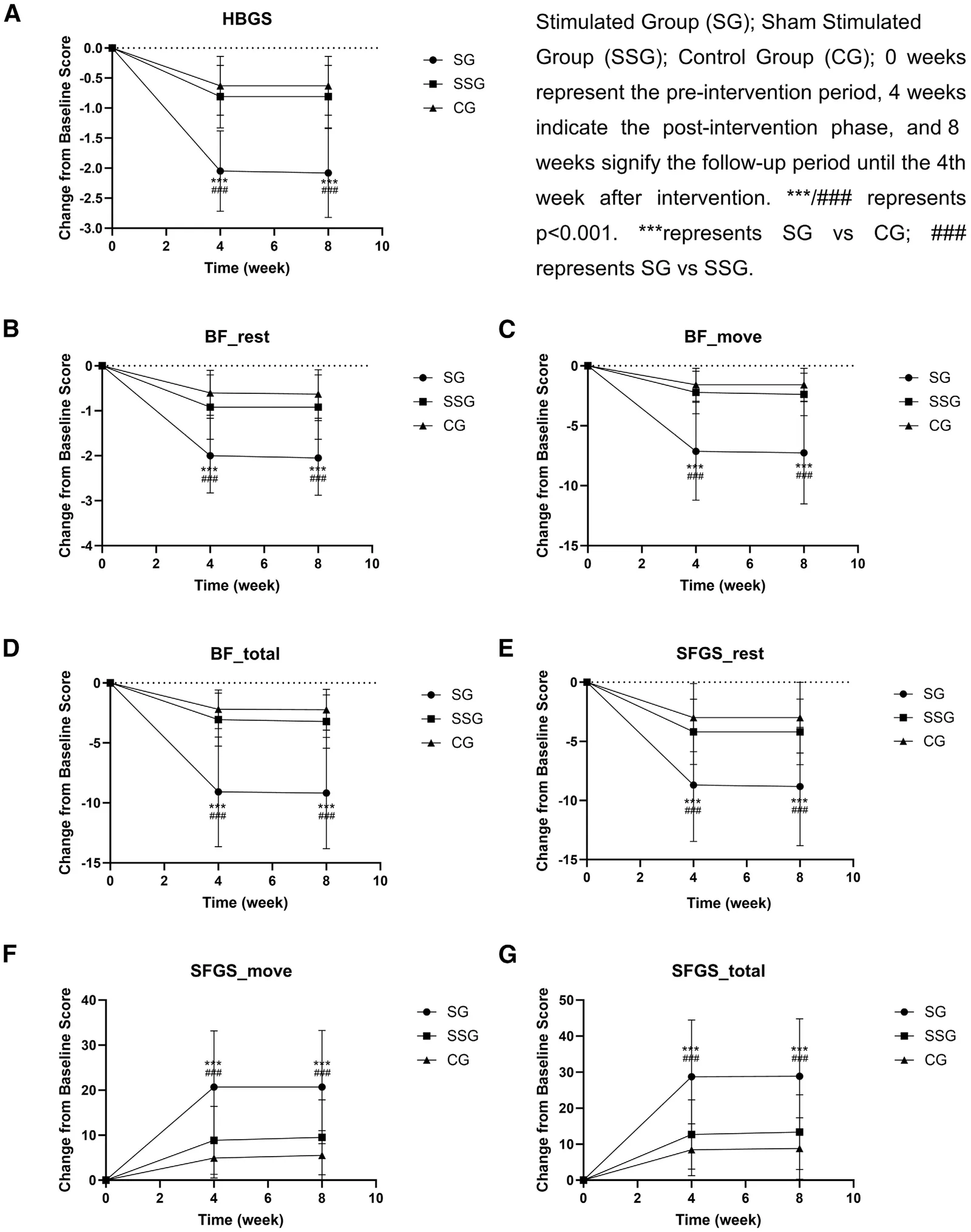
Primary and secondary efficacy endpoints. (A) HBGS; (B) BF_rest; (C) BF_move; (D) BF_total; (E) SFGS_rest; (F) SFGS_move; (G) SFGS_total. Week 0 represents the pre-intervention period, week 4 indicates the post-intervention phase, and week 8 represents the 4-week follow-up after intervention. *P < .001 for SG vs CG; ###*P* < .001 for SG vs SSG. BF_move = Burres–Fisch movement score, BF_rest = Burres–Fisch resting score, BF_total = Burres–Fisch total score, CG = control group, HBGS = House-Brackmann grading scale, SFGS_move =Sunnybrook facial grading system movement score, SFGS_rest = Sunnybrook facial grading system resting score, SFGS_total = Sunnybrook facial grading system total score, SG = stimulated group, SSG = sham stimulated group.

### 3.2. Quality of life

Following a 4-week intervention, all 3 groups exhibited varying degrees of FDIp score improvements, with the SG group demonstrating the most significant increase (*P* < .05). Additionally, FDIs scores decreased to varying extents, particularly in the SG group (*P* < .05). This pattern remained consistent until the 8th week (Table 2).

### 3.3. Dysphagia

Table 2 shows that all 3 groups exhibited improved FOIS scores following the intervention. However, no statistically significant differences were observed among the 3 groups at either the 4th- or 8th-week time points (*P* > .05) (Table 2). Individuals with facial paralysis may experience oral food retention or unintentional spillage while eating due to diminished facial sensation and muscle strength. Tracking the proportion of patients exhibiting complete resolution of symptoms (no residue) at various time points provides insight into facial function recovery across different treatment modalities in 3 separate cohorts. At baseline, the proportion of patients with no residue was 2.5% in all 3 groups. After 4 weeks of intervention, this increased to 67.5% in the SG group, 45.0% in the SSG group, and 35.0% in the CG group. There were statistically significant differences between the SG, SSG, and CG groups (*P* < .05). However, no statistical significance was observed between the SSG and CG (*P* > .05) groups (Table 3).

**Table 3:** Food residue in the oral cavity on the side of facial paralysis.

No. food residue	SG (pre) n = 40	SG (post) n = 40	SSG (pre) n = 40	SSG (post) n = 40	CG (pre) n = 40	CG (post) n = 40
No residue	1 (2.5%)	27 (67.5%)^a^	1 (2.5%)	18 (45.0%)^b^	1 (2.5%)	14 (35.0%)^b^
Mild residue	18 (45.0%)	13(32.5%)	18 (45.0%)	15(37.5%)	14 (35.0%)	15 (37.5%)
Moderate residue	13 (32.5%)	0 (0.0%)	16(40%)	7 (17.5%)	19 (47.5%)	7 (17.5%)
Severe residual	8 (20.0%)	0(0.0%)	5(12.5%)	0(0.0%)	6 (15.0%)	4(10.0%)
Total	40 (100.0%)	40(100.0%)	40 (100.0%)	40 (100.0%)	40 (100.0%)	40 (100.0%)

The chi-square test was utilized for both within-group and between-group comparisons. The primary comparison focuses on the proportion of individuals who have completely recovered, both within and between groups. According to the letters a and b, 3 groups are used to indicate statistical differences. If 2 groups have the same letter, it means there is no statistical difference between them; while if the letters between 2 groups are different (a, b), it implies that there is a significant statistical difference between them (*P* < .05).

CG = control group, SG = stimulated group, SSG = sham stimulated group.

## 4. Discussion

Our findings indicate that KT is effective in treating CFP. Compared to conventional rehabilitation therapy alone, combining KT and conventional rehabilitation therapy yields superior therapeutic outcomes. Additionally, a comparison between SG and SSG data shows that SG outperforms SSG in improving both dynamic and static facial nerve function, with no significant difference observed between SSG and CG. These results suggest that KT efficacy may be linked to the tension applied during taping. However, whether varying levels of tension have differential effects on the efficacy of CFP treatment remains unclear, warranting further investigation.

Current research on CFP remains limited, with a notable absence of purpose-built evaluation scales specifically designed for this unique type of brain-related facial movement disorder. Emerging evidence from recent investigations validates the clinical utility of both the HBGS and SFGS in the assessment of CFP.^[11]^ However, relying on a single scale presents significant limitations. Therefore, we employed 3 commonly utilized facial palsy scales (HBGS, SFGS, and BF) to evaluate treatment effectiveness, aiming to enhance the reliability of our research findings. The reliability and validity of these scales have been comprehensively validated, with rigorous statistical confirmation of their consistent performance across facial paralysis subtypes, etiologies, and age groups.^[11,14,15,23]^ In addition to HBGS, SFGS, and BF, independently analyze static and dynamic facial features and assign them weights. Thus, to assess the effects of facial paralysis treatment, we presented data on static and dynamic facial symmetry from SFGS and BF, allowing us to further explore the differential impact of KT on improving patients’ static and dynamic facial function. The findings revealed that KT enhanced patients’ static facial symmetry and significantly improved their dynamic symmetry (Table 2). Moreover, through longitudinal analysis (2-way repeated-measures ANOVA), we observed that the therapeutic effects in the SG group remained stable from post-intervention at week 4 (*T*1) to the 8-week follow-up (*T*2) (within-group *T*1 vs *T*2: *P* > .05) and consistently outperformed the SSG and CG groups (*P* < .001). This time-dependent analysis not only validates the immediate efficacy of KT intervention but also provides robust evidence for its sustained benefits in mid-term rehabilitation, supporting its long-term clinical utility in CFP management.

The mechanism of KT treatment for CFP is not yet fully understood. Some studies suggest that KT may ameliorate functional impairment by enhancing muscle strength. A meta-analysis demonstrated its efficacy in increasing isokinetic muscle strength in patients with osteoarthritis.^[24,25]^ However, some research findings are inconclusive, with some suggesting that KT may not increase muscle strength.^[26]^ On the other hand, most studies agree that KT enhances proprioceptive input by increasing skin sensory input and stimulating joint mechanoreceptors, stimulating neural tissue remodeling in the brain, thereby improving muscle strength control.^[27–29]^ This aligns with our hypothesis that KT improves facial function by heightening proprioception in facial muscles and tendons. KT generates its own tension and produces a retraction force through the tape. Increasing the tape’s tension within a specific range can enhance stability and support for local tissues. However, excessive tension may diminish the benefits of muscle taping for certain clinical indications, as recent studies have shown.^[30,31]^ Therefore, the optimal level of applied tension remains to be determined and requires further investigation in future studies.

The selection of tension-free KT as the sham intervention was driven by the need to balance methodological rigor and ecological validity. Unlike non-stretchable tapes, which differ markedly from KT in texture, elasticity, and skin adherence, tension-free KT preserves the material properties of therapeutic KT while eliminating active tension. This design aimed to minimize unblinding risks – particularly for patients familiar with KT – and reduce performance bias caused by sensory discrepancies. For instance, studies on cervical proprioception in athletes with mechanical neck pain,^[32]^ isokinetic quadriceps torque in knee osteoarthritis,^[33]^ chronic low back pain,^[34]^ and delayed-onset muscle soreness^[35]^ have similarly employed tension-free KT to isolate tension-specific effects without compromising blinding. However, this approach introduces a potential limitation: the inherent elasticity of KT may still exert subtle mechanical or proprioceptive effects, even when applied without tension. This contrasts with sham protocols using non-stretchable tapes, which eliminate elasticity-related confounders but fail to replicate KT’s tactile experience. These collective observations underscore the complexity of disentangling biomechanical effects from placebo responses in KT research. Future studies should explore hybrid sham designs (e.g., non-stretchable tape mimicking KT patterns) to better isolate therapeutic mechanisms while maintaining blinding fidelity. Such refinements could address the limitations inherent in both tension-free and non-stretchable sham protocols, advancing the standardization of KT trials.

Our study employed a multi-domain evaluation protocol to capture the complex clinical manifestations of CFP. In addition to primary facial function scales (HBGS, SFGS, and BF), we incorporated the FOIS and FRC to specifically assess dysphagia severity and oral-phase swallowing efficiency. The facial nerve contributes to the oropharyngeal phase of swallowing by innervating the buccinator, perioral, digastricus posterior, and stylohyoid muscles.^[36]^ Weakness of the facial muscles can impair the oral phase of swallowing, leading to reduced mastication and bolus control function, oral residue, and drooling.^[37]^ Jong-Bae et al suggested that strengthening facial muscles can improve the oral phase of swallowing.^[37]^ Therefore, we conducted additional observations to evaluate the effects of KT application concerning dysphagia improvement in stroke patients. Notably, the SS failed to demonstrate clinical superiority in swallowing functional outcomes (FOIS) (Table 2). This phenomenon may be mechanistically explained by 2 factors: while current interventions effectively targeted oral-phase functional remodeling (evidenced by 67.5% FRC improvement, Table 3), their influence on pharyngeal-phase biomechanical regulation – particularly cricopharyngeal opening dynamics and laryngeal elevation velocity – remained suboptimal. This finding aligns with the observations reported by Jong-Bae et al.^[38]^ Concurrent administration of foundational swallowing therapies (e.g., Shaker exercises and transcutaneous electrical stimulation) across all groups likely contributed to comparable FOIS score improvements (*T*1 vs *T*0, *P* < .05), thereby masking intergroup differences. Although the SG group exhibited a marginally favorable trend, statistical significance was not achieved. Collectively, these findings underscore the imperative to incorporate pharyngeal-specific modalities (e.g., surface electromyography biofeedback) and longitudinal follow-up in future investigations to dissect the temporal interplay between facial motor recovery and swallowing rehabilitation.

Notably, none of the 116 patients in our study achieved complete recovery (HBGS = 1), which differs significantly from the typical progression seen in peripheral facial palsy. While patients with peripheral facial palsy typically experience partial or full recovery within a month, this pattern does not apply to CFP patients. This discrepancy may be attributed to the fact that CFP caused by stroke is primarily associated with intracranial lesions requiring stronger stimulation or a longer cumulative period for brain remodeling or functional area replacement. In addition, no serious adverse effects were observed in our study.

This study has several limitations. First, electrophysiological indicators, such as electromyography, were not utilized, and the outcome measures were restricted to HBGS, SFGS, and BF, which may introduce some subjectivity. Second, the 4-week follow-up period, while demonstrating short-term efficacy, cannot confirm long-term benefits. Extended follow-ups (≥6 months) are needed to assess sustained recovery in chronic stages. Finally, the lack of stratification by stroke duration (e.g., acute vs subacute) may confound treatment responses, despite enrollment within 6 months poststroke. Future studies should stratify by stroke duration to clarify KT’s time-sensitive efficacy.

## 5. Conclusion

In conclusion, this study demonstrates that KT applied with specific tension constitutes an effective adjunct to conventional rehabilitation for patients with CFP. The principal findings indicate that beyond significantly enhancing the recovery of static and dynamic facial symmetry, KT sustains these functional gains for at least 4 weeks following treatment cessation. Furthermore, the intervention yielded notable improvements in oral-phase swallowing function, evidenced by superior residue clearance, and promoted clinically meaningful gains in quality of life. Critically, the absence of significant therapeutic effects in the SSG, which received KT without appropriate tension, confirms that the observed benefits are attributable to proprioceptive input generated by mechanical stretch. This finding underscores the necessity of correct tension application for KT efficacy. Collectively, these results support the integration of tension-based KT into standardized CFP rehabilitation protocols to optimize motor recovery and functional outcomes. Future investigations should prioritize elucidating the underlying neurophysiological mechanisms and refining application parameters to maximize clinical benefits.

## Author contributions

**Conceptualization:** Xue Li.

**Data curation:** Jiaojiao Wang.

**Investigation:** Hua Xiao, Shuangjiang Li, Siyi Wang, Qing Liu.

**Methodology:** Jiaojiao Wang.

**Project administration:** Xue Li.

**Resources:** Shuangjiang Li.

**Software:** Jiaojiao Wang.

**Validation:** Hua Xiao.

**Writing – original draft:** Xue Li.

**Writing – review & editing:** Chenlan Shao.


