# Supplementary material for: Efficacy of Kinesio tape for central facial paralysis: A prospective, randomized sham-controlled, single-blinded clinical trial
Source: Medicine (Baltimore). 2026 Oct 9;105(41):e51043. doi: 10.1097/MD.0000000000051043 (PMC13657581; doi:10.1097/MD.0000000000051043)
Supplement: Supplementary file 1 [file medi-105-e51043-s001.docx]

**Supplementary Table Facial Improvement Outcomes at Baseline (Week 0), Post-intervention (Week 4), and 8-Week Follow-up (Week 8)**

| **Time** | **Group** | **n** | **HBGS**  **(Mean ± SD)** | **Simple Effects Analysis of Group** | | **Repeated-Measures ANOVA** | | |
| --- | --- | --- | --- | --- | --- | --- | --- | --- |
|  |  |  |  | **F** | ***p*** | **F** | ***p*** | **Partial η²** |
| T0 | SG | 39 | 4.38±0.71 | 0.413 | 0.663 |  |  |  |
|  | SSG | 37 | 4.35±0.63 |  |  |  |  |  |
|  | CG | 40 | 4.25±0.71 |  |  |  |  |  |
| T1 | SG | 39 | 2.33±0.66^a^ | 48.636 | ＜0.001 |  |  |  |
|  | SSG | 37 | 3.54±0.56^b^ |  |  |  |  |  |
|  | CG | 40 | 3.63±0.70^b^ |  |  |  |  |  |
| T2 | SG | 39 | 2.31±0.69^a^ | 48.943 | ＜0.001 |  |  |  |
|  | SSG | 37 | 3.54±0.56^b^ |  |  |  |  |  |
|  | CG | 40 | 3.63±0.70^b^ |  |  |  |  |  |
| Group effect |  |  |  |  |  | 23.582 | ＜0.001 | 0.294 |
| Time effect |  |  |  |  |  | 458.501 | ＜0.001 | 0.802 |
| Time x Group |  |  |  |  |  | 69.543 | ＜0.001 | 0.552 |
|  |  |  | **BF_rest**  **(Mean ± SD)** |  | |  |  |  |
| T0 | SG | 39 | 3.10±0.85 | 1.171 | 0.314 |  |  |  |
|  | SSG | 37 | 3.30±0.78 |  |  |  |  |  |
|  | CG | 40 | 3.03±0.77 |  |  |  |  |  |
| T1 | SG | 39 | 1.10±0.75^a^ | 36.352 | ＜0.001 |  |  |  |
|  | SSG | 37 | 2.38±0.76^b^ |  |  |  |  |  |
|  | CG | 40 | 2.43±0.81^b^ |  |  |  |  |  |
| T2 | SG | 39 | 1.05±0.76^a^ | 38.421 | ＜0.001 |  |  |  |
|  | SSG | 37 | 2.38±0.76^b^ |  |  |  |  |  |
|  | CG | 40 | 2.40±0.81^b^ |  |  |  |  |  |
| Group effect |  |  |  |  |  | 20.768 | ＜0.001 | 0.269 |
| Time effect |  |  |  |  |  | 325.183 | ＜0.001 | 0.742 |
| Time x Group |  |  |  |  |  | 43.229 | ＜0.001 | 0.433 |
|  |  |  | **BF_move**  **(Mean ± SD)** |  |  |  |  |  |
| T0 | SG | 39 | 10.95±5.56 | 1.674 | 0.192 |  |  |  |
|  | SSG | 37 | 10.49±4.05 |  |  |  |  |  |
|  | CG | 40 | 9.18±3.56 |  |  |  |  |  |
| T1 | SG | 39 | 3.82±3.36^a^ | 21.006 | ＜0.001 |  |  |  |
|  | SSG | 37 | 8.27±3.30^b^ |  |  |  |  |  |
|  | CG | 40 | 7.60±3.10^b^ |  |  |  |  |  |
| T2 | SG | 39 | 3.69±3.39^a^ | 21.289 | ＜0.001 |  |  |  |
|  | SSG | 37 | 8.11±3.34^b^ |  |  |  |  |  |
|  | CG | 40 | 7.60±3.10^b^ |  |  |  |  |  |
| Group effect |  |  |  |  |  | 6.563 | 0.002 | 0.104 |
| Time effect |  |  |  |  |  | 208.469 | ＜0.001 | 0.648 |
| Time x Group |  |  |  |  |  | 48.215 | ＜0.001 | 0.460 |
|  |  |  | **BF_total**  **(Mean ± SD)** |  |  |  |  |  |
| T0 | SG | 39 | 14.00±6.17 | 1.435 | 0.243 |  |  |  |
|  | SSG | 37 | 13.73±4.50 |  |  |  |  |  |
|  | CG | 40 | 12.23±4.13 |  |  |  |  |  |
| T1 | SG | 39 | 4.92±3.78^a^ | 27.750 | ＜0.001 |  |  |  |
|  | SSG | 37 | 10.68±3.63^b^ |  |  |  |  |  |
|  | CG | 40 | 10.03±3.74^b^ |  |  |  |  |  |
| T2 | SG | 39 | 4.82±3.82^a^ | 27.854 | ＜0.001 |  |  |  |
|  | SSG | 37 | 10.51±3.69^b^ |  |  |  |  |  |
|  | CG | 40 | 10.00±3.72^b^ |  |  |  |  |  |
| Group effect |  |  |  |  |  | 9.375 | ＜0.001 | 0.142 |
| Time effect |  |  |  |  |  | 277.541 | ＜0.001 | 0.711 |
| Time x Group |  |  |  |  |  | 56.618 | ＜0.001 | 0.501 |
|  |  |  | **SFGS_rest**  **(Mean ± SD)** |  |  |  |  |  |
| T0 | SG | 39 | 13.46±5.02 | 0.311 | 0.734 |  |  |  |
|  | SSG | 37 | 13.51±4.69 |  |  |  |  |  |
|  | CG | 40 | 12.75±4.66 |  |  |  |  |  |
| T1 | SG | 39 | 4.77±2.99^a^ | 17.716 | ＜0.001 |  |  |  |
|  | SSG | 37 | 9.32±4.28^b^ |  |  |  |  |  |
|  | CG | 40 | 9.75±4.80^b^ |  |  |  |  |  |
| T2 | SG | 39 | 4.64±3.08^a^ | 18.459 | ＜0.001 |  |  |  |
|  | SSG | 37 | 9.32±4.28^b^ |  |  |  |  |  |
|  | CG | 40 | 9.75±4.80^b^ |  |  |  |  |  |
| Group effect |  |  |  |  |  | 7.940 | 0.001 | 0.123 |
| Time effect |  |  |  |  |  | 233.275 | ＜0.001 | 0.674 |
| Time x Group |  |  |  |  |  | 25.775 | ＜0.001 | 0.313 |
|  |  |  | **SFGS_move**  **(Mean ± SD)** |  |  |  |  |  |
| T0 | SG | 39 | 71.56±14.49 | 0.957 | 0.387 |  |  |  |
|  | SSG | 37 | 72.00±11.23 |  |  |  |  |  |
|  | CG | 40 | 75.10±11.08 |  |  |  |  |  |
| T1 | SG | 39 | 92.26±4.77^a^ | 20.697 | ＜0.001 |  |  |  |
|  | SSG | 37 | 80.86±11.11^b^ |  |  |  |  |  |
|  | CG | 40 | 80.00±10.91^b^ |  |  |  |  |  |
| T2 | SG | 39 | 92.23±4.71^a^ | 20.031 | ＜0.001 |  |  |  |
|  | SSG | 37 | 81.51±11.08^b^ |  |  |  |  |  |
|  | CG | 40 | 80.60±10.51 |  |  |  |  |  |
| Group effect |  |  |  |  |  | 7.053 | 0.001 | 0.111 |
| Time effect |  |  |  |  |  | 189.742 | ＜0.001 | 0.627 |
| Time x Group |  |  |  |  |  | 30.385 | ＜0.001 | 0.350 |
|  |  |  | **SFGS_total**  **(Mean ± SD)** |  |  |  |  |  |
| T0 | SG | 39 | 58.74±18.95 | 0.959 | 0.387 |  |  |  |
|  | SSG | 37 | 58.30±14.63 |  |  |  |  |  |
|  | CG | 40 | 62.90±14.68 |  |  |  |  |  |
| T1 | SG | 39 | 87.49±6.32^a^ | 24.091 | ＜0.001 |  |  |  |
|  | SSG | 37 | 71.00±13.66^b^ |  |  |  |  |  |
|  | CG | 40 | 71.33±14.27^b^ |  |  |  |  |  |
| T2 | SG | 39 | 87.59±6.40^a^ | 23.812 | ＜0.001 |  |  |  |
|  | SSG | 37 | 71.65±13.69^b^ |  |  |  |  |  |
|  | CG | 40 | 71.73±14.12^b^ |  |  |  |  |  |
| Group effect |  |  |  |  |  | 8.850 | ＜0.001 | 0.135 |
| Time effect |  |  |  |  |  | 234.954 | ＜0.001 | 0.675 |
| Time x Group |  |  |  |  |  | 31.594 | ＜0.001 | 0.359 |

Note: Stimulated Group (SG); Sham Stimulated Group (SSG); Control Group (CG); House-Brackmann Grading Scale (HBGS); Burres-Fisch Grading System (BF); Sunnybrook Facial Grading System(SFGS); T0, T1, and T2 represent the outcome assessment values at baseline (pre-intervention, week 0), immediately post-intervention (week 4), and follow-up (week 8), respectively. All the data have a parametric distribution presented as mean ±SD. If two groups have the same letter (b, b), it means there is no statistical difference between them; while if the letters between two groups are different (a, b), it implies that there is a significant statistical difference between them (*p*<0.05). Partial η²: Effect size thresholds: 0.01 (small), 0.06 (medium), 0.14 (large).
